# Untapping the Health Enhancing Potential of Vigorous Intermittent Lifestyle Physical Activity (VILPA): Rationale, Scoping Review, and a 4-Pillar Research Framework

**DOI:** 10.1007/s40279-020-01368-8

**Published:** 2020-10-26

**Authors:** Emmanuel Stamatakis, Bo-Huei Huang, Carol Maher, Cecilie Thøgersen-Ntoumani, Afroditi Stathi, Paddy C. Dempsey, Nathan Johnson, Andreas Holtermann, Josephine Y. Chau, Catherine Sherrington, Amanda J. Daley, Mark Hamer, Marie H. Murphy, Catrine Tudor-Locke, Martin J. Gibala

**Affiliations:** 1 School of Health Sciences, Charles Perkins Centre, The University of Sydney, Faculty of Medicine and Health, Hub D17, L6 West, Sydney, NSW Australia; 2 Allied Health and Human Performance, University of South Australia, Adelaide, Australia; 3 Physical Activity and Well-Being Research Group, School of Psychology, Curtin University, Perth, Australia; 4 School of Sport, Exercise and Rehabilitation Sciences, University of Birmingham, Birmingham, UK; 5 Physical Activity and Behavioural Epidemiology Laboratories, Baker Heart and Diabetes Institute, Melbourne, Australia; 6 National Research Centre for the Working Environment (NRCWE), Copenhagen, Denmark; 7 Department of Health Systems and Populations, Macquarie University, Sydney, Australia; 8 Institute of Musculoskeletal Health, University of Sydney, Sydney School of Public Health, Faculty of Medicine and Health, Sydney, Australia; 9 School of Sport, Exercise and Health Sciences, Loughborough University, Loughborough, UK; 10 Institute Sport Exercise Health, Faculty Medical Sciences, University College London, London, UK; 11 Doctoral College, Ulster University, Newtownabbey, Co Antrim BT37 0QB Northern Ireland, UK; 12 Department of Kinesiology, College of Health and Human Services, University of North Carolina at Charlotte, Charlotte, NC USA; 13 Department of Kinesiology, McMaster University, Hamilton, ON Canada

## Abstract

**Abstract:**

Recently revised public health guidelines acknowledge the health benefits of regular intermittent bouts of vigorous intensity incidental physical activity done as part of daily living, such as carrying shopping bags, walking uphill, and stair climbing. Despite this recognition and the advantages such lifestyle physical activity has over continuous vigorous intensity structured exercise, a scoping review we conducted revealed that current research in this area is, at best, rudimentary. Key gaps include the absence of an empirically-derived dose specification (e.g., minimum duration of lifestyle physical activity required to achieve absolute or relative vigorous intensity), lack of acceptable measurement standards, limited understanding of acute and chronic (adaptive) effects of intermittent vigorous bouts on health, and paucity of essential information necessary to develop feasible and scalable interventions (e.g., acceptability of this kind of physical activity by the public). To encourage collaboration and research agenda alignment among groups interested in this field, we propose a research framework to further understanding of vigorous intermittent lifestyle physical activity (VILPA). This framework comprises four pillars aimed at the development of: (a) an empirical definition of VILPA, (b) methods to reliably and accurately measure VILPA, (c) approaches to examine the short and long-term dose–response effects of VILPA, and (d) scalable and acceptable behavioural VILPA-promoting interventions.

**Graphic Abstract:**

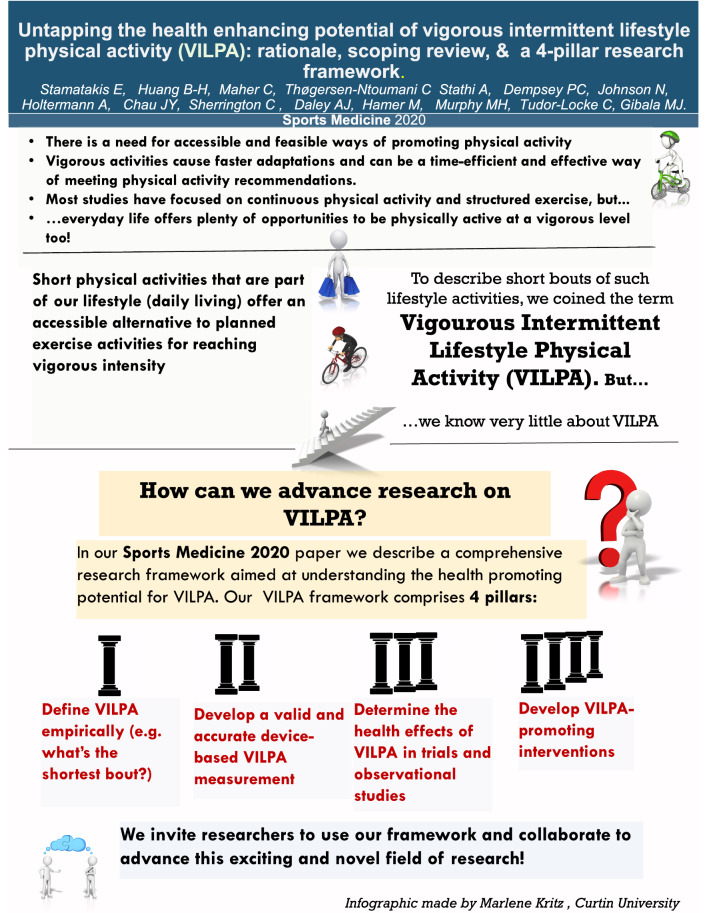

**Electronic supplementary material:**

The online version of this article (10.1007/s40279-020-01368-8) contains supplementary material, which is available to authorized users.

## Key Points

**Table:** 

Vigorous Intermittent Lifestyle Physical Activity (VILPA) is characterised by brief bouts of incidental physical activity that are done during activities of daily living.
Although VILPA may be more feasible than structured vigorous exercise in some population groups, it is an unexplored aspect of physical activity.
The research framework we propose covers development of an empirical VILPA definition, improvements in the free-living VILPA measurement, studies to better understand its dose–response effects on health, and the development of scalable and acceptable behavioural VILPA-promoting interventions.

## Introduction

Physical inactivity, or insufficient physical activity (PA), is a major cause of non-communicable chronic disease responsible for at least 5 million premature deaths per year [1]. Global data show that physical inactivity (defined as not meeting the World Health Organization (WHO) aerobic PA recommendation [2]) has increased by 15% between 2001 and 2016 in high income countries [3, 4]. At the individual level, perceived lack of time and low priority are commonly cited reasons for physical inactivity [5].

Vigorous PA (> 6 absolute MET or ≥ 14 or > 15 [6, 7] on perceived effort/according to the self-reported relative intensity Borg Rating Scale) is a potentially time-efficient strategy for accumulating PA. In addition to time economy, vigorous intensity PA can elicit additional health enhancing responses compared to equivalent volumes of moderate and low intensities (i.e., < 6 METs) [8, 9]. However, regular participation in continuous and structured vigorous intensity PA that is confined to leisure-time can be logistically demanding or unappealing for most of the population due to reasons other than lack of time [5, 10], e.g., need for preparation and travelling to exercise facilities, lack of exercise skills and confidence, and discomfort associated with high exertion. Furthermore, it can be challenging for people with lower levels of fitness, a state which often clusters with other established biomedical risk factors [11, 12] such as hypertension, type 2 diabetes, and overweight/obesity. These conditions affect the majority of the middle-aged and older population in high income countries [13] and are becoming increasingly prevalent in low- and middle-income countries [14]. Population level estimates highlight the poor feasibility of vigorous intensity structured exercise. For example, only about 20% of adults aged 40–65 years report doing *any* vigorous exercise for at least 15 continuous minutes a month [15]. Mean accelerometry-determined average time spent on high intensity PA is as low as 42 s per day for US adults [16]. These very low participation rates have resulted in researchers, clinicians, and policy makers typically paying very little attention to the contribution of vigorous intensity PA, especially when performed as part of daily living *outside* the leisure-time domain.

Lifestyle (incidental) PA encompasses activities of daily living that require little or no dedicated time commitment and are *not* performed specifically for the purpose of leisure or health/fitness benefits [17]. Lifestyle PA may have feasibility advantages, as most barriers to structured exercise participation—namely lack of time and motivation, costs, poor access to facilities and a low fitness level are less likely to be present.

**Fig. 1:**
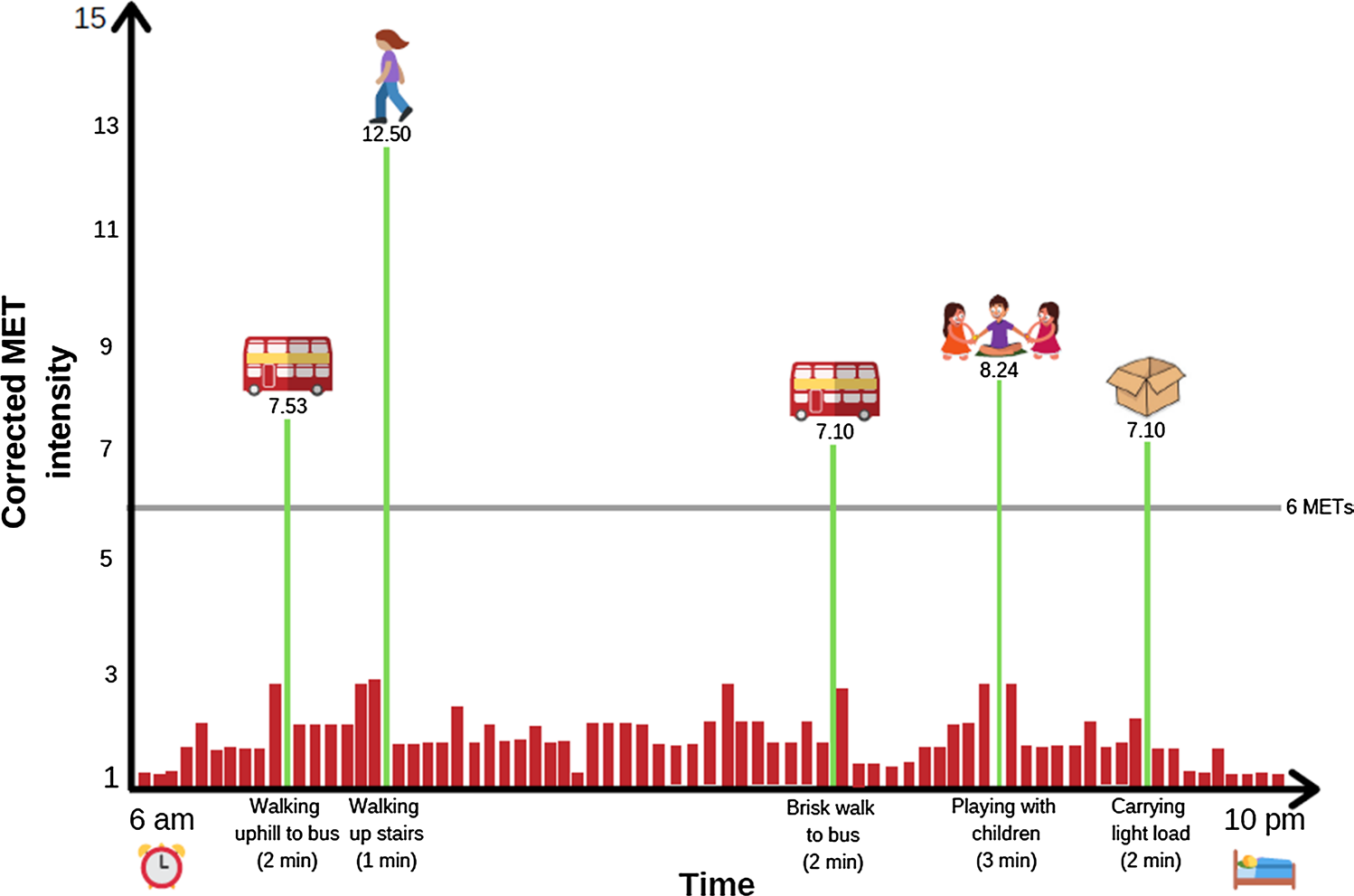
An example of a day characterized by a 10 min/bout pattern of vigorous intermittent lifestyle physical activity (VILPA). MET values reflect relative intensity as described in Martenstyn et al. 2019 [55]. Figure reproduced under CC BY-NC 4.0 license from Stamatakis E et al. Br J Sports Med 2019;53:1137–1139 [56]

Prompted by changes in the 2018 US [18] and 2019 UK [19] PA guidelines, we have recently proposed a new paradigm which would allow vigorous PA to be more accessible to people who are currently inactive through the regular accumulation of vigorous intermittent lifestyle physical activity (VILPA) [17]. This approach, which is aligned with previous calls proposing a shift towards integrated and multi-dimensional PA paradigms [20, 21], refers to brief intermittent bursts of vigorous intensity PA embedded incidentally/secondary to regular activities of daily living. Examples of VILPA include bouts of stair climbing, carrying children or groceries for 50–100 m, and maximizing walking pace for a short distance (e.g., 100–200 m) to reach vigorous intensity [17]. In the absence of empirical standards and for the purposes of this article, we broadly define a VILPA bout as a single session lasting for up to 5 min of relative (Borg Rating Scale ≥ 14 or > 15) [6, 7] or absolute (> 6 MET) vigorous PA that occurs during activities of daily living or during other lifestyle PA. A hypothetical VILPA pattern for an otherwise physically inactive middle-aged man is presented in Fig. 1. It portrays 10 min of VILPA per day accumulated through short bouts (lasting up to 3 min in this example). Such a pattern requires little additional time commitment or planning (compared to, e.g., vigorous leisure-time PA), since all activities are part of daily living, and in some cases could even shorten the time needed to perform the primary activity. Repeated daily, such a quantum of VILPA could produce 65% of recommended weekly PA [22]. The Australian Institute of Health and Welfare has estimated that if all adults increased their PA by such an amount there would be a 13% decrease in chronic disease burden compared to no moderate to vigorous PA [23].

**Fig. 2:**
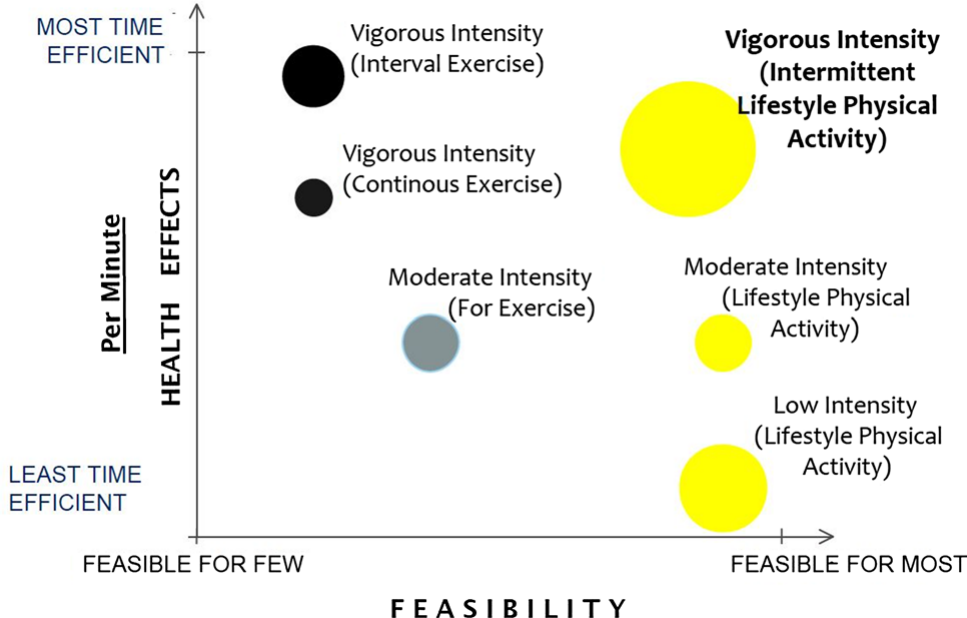
Hypothetical comparison of the different physical activity components in terms of time efficiency and theoretical feasibility. The illustration is based on the authors’ rough appraisal of the evidence base to date and is not meant to represent a general consensus on the feasibility or research gaps surrounding each physical activity class. *Yellow*: inherent part of daily living; *grey*: requires planning; *black*: requires planning, high levels of motivation, and sometimes direct supervision/skills/costs. The size of the bubbles illustrates gaps in knowledge (larger: we know less)

Adopting a daily pattern of VILPA is theoretically akin to practicing popular exercise regimens that also emphasize non-continuous vigorous intensity exercise, such as high intensity interval training (HIIT). HIIT is a structured exercise approach that improves key cardiometabolic health markers, particularly cardiorespiratory fitness (CRF) [24]. However, HIIT may be less accessible to large parts of the population [25], especially those who are the least physically active and fit [26]. Incorporating HIIT principles into a more accessible form of daily vigorous PA is a “missing link” in research and practice. Although VILPA (or equivalent concepts) is promising in terms of theoretical population-based feasibility and time-efficiency, it is one of the least researched aspects of PA (Fig. 2).

Informed by a systematic scoping review conducted to map the emerging research area of VILPA and identify critical research gaps, the aim of this Leading Article is to highlight future research directions on this topic across four pillars: (a) an empirical definition of VILPA, (b) methods to reliably and accurately measure VILPA, (c) approaches to examine the short and long-term dose–response effects of VILPA, and (d) scalable and acceptable behavioural VILPA-promoting interventions.

## Mapping Relevant Research to Date—A Scoping Review

We searched three databases (PubMed, CINAHL, and Embase) to identify any peer-reviewed literature (original research or reviews) relevant to VILPA. As no standardized terminology exists to describe what is herein referred to as VILPA, we used terms that reflect roughly the same concept as VILPA, such as “high intensity incidental PA” [17], “exercise snacks” [27, 28], and “snacktivity”. Electronic Supplementary Material Appendix S1 describes the methods of the scoping review including the search strategy. We identified four observational cross-sectional studies (two in adults, two in children), four intervention studies (all in adults), and one narrative review. Electronic Supplementary Material Appendix S2 describes the full set of the scoping review results, including data extraction tables with full study details (Tables S1–2). In brief, our scoping review identified limited literature in relation to the four research framework pillars we examine: no standardized or empirically derived definition for any VILPA-related concepts exists. In addition, no measurement criteria specific to VILPA have been developed to date. Most observational studies have used waist-worn accelerometry to quantify short bouts. However, they have used 1-min sampling epochs which may miss shorter VILPA bouts, such shorter bouts of stair climbing or walking uphill. Existing physiological VILPA-relevant interventions [27, 29] are proof-of-concept or pilot in nature. Despite these limitations, the available studies suggest that brief bouts of regular VILPA (stair climbing or fast walking) can potentially improve cardiorespiratory fitness with a total volume of around 10 min of non-consecutive bouts per day. With the exception of a few stair-climbing programs, we did not identify interventions aimed at specifically promoting VILPA, or related kinds of PA. We expand on the existing literature in all four proposed framework pillars we describe below.

## Future Research Needs—A 4-Pillar Framework

### Pillar 1: Definition

Until recently, major authorities, including the WHO [2], recommended that adults should perform aerobic PA in continuous bouts lasting at least 10 min. By ignoring shorter bouts, such recommendations assigned *no health value* to intermittent bouts of lifestyle PA, regardless of intensity [2]. The 2018 US [18, 30] and 2019 UK [19] PA guidelines eventually removed the minimum bout duration requirement. The UK 2019 adult guidelines now explicitly recommend “…shorter durations of very vigorous intensity activity (such as sprinting or stair climbing)”. Similarly, the US guidance now encourages PA of any duration to be included in an accumulated total daily volume. Such changes in the guidelines were based on *absence of evidence* (to support a minimum bout duration of 10 min) rather than direct evidence of the health enhancing properties of shorter and intermittent bouts. With the exception of certain HIIT protocols involving very short exercise bursts (e.g., 6 s), the minimum specification of a “short bout” of health enhancing PA in still unknown, particularly in the context of everyday life and outside the realm of purposeful exercise. A recent systematic review [31] concluded that splitting a continuous exercise bout into shorter bouts of equivalent total volume interspersed across a day does not diminish the potential to provide cardiometabolic health benefits. While that review supports the overall principle of PA accumulation, it did not shed light on the minimum bout length required, as only one of 19 identified studies examined bouts < 10 min in duration [31].

One of the first requirements for advancing the VILPA research agenda is to empirically and operationally define what future studies should consider a “VILPA bout” in terms of minimum time needed to reach absolute and relative vigorous intensity during activities of daily living. The relative intensity aspect of such a definition will likely be age-specific to incorporate functional status and relative fitness. Examples of future research needs include studies to determine the minimal duration required for common lifestyle physical activities (e.g., walking on a flat or inclined surface, ascending stairs, carrying shopping bags) performed to reach vigorous intensity defined objectively (e.g., ~ 80% of age-predicted heart rate maximum, or > 6 MET) or subjectively (e.g., Borg Rating ≥ 14 or > 15) [6, 7]. To maximize public health relevance, the empirical definition of VILPA should be generalizable to those population groups most in need of PA, including those who are physically inactive and time-limited, have low fitness levels, and have other disease risk factors.

### Pillar 2: Measurement

Observational cohort studies with questionnaire-based PA measures account for most evidence used in PA guidelines development globally [2, 19, 22, 32]. The inherent inability of questionnaires to capture short and intermittent PA bouts preventing studies from examining associations of VILPA with health outcomes is another key reason why VILPA has not been a mainstream research priority. Wearable accelerometer-based technologies are now more mainstream [17] in large scale observational studies. For example, accelerometers worn 24 h a day for a whole week have considerably better potential than recall-based questionnaires to capture the granularity of VILPA performance patterns [33].

Measuring capacity of accelerometers varies by accelerometer placement. For example, waist-mounted accelerometers can collect frequency, duration, and mode information as well as speed of pace-based indicators of intensity, but cannot capture intensity modulation due to changes in surface gradient (e.g., walking uphill or climbing stairs). Additionally, the existence of a dozen different waist-mounted accelerometry standards (cut-off points) to define vigorous PA [34] is challenging for researchers. Thigh-mounted accelerometers, which are capable of differentiating between postures and PA modes (e.g., stair climbing [33]), have no specific empirically-derived standards to capture any bouts of vigorous intensity using the accelerometer signal and cannot capture the intensity effects of walking on a gradient or carrying loads. With relatively few exceptions [35, 36], the opportunities for PA intensity classification offered by existing walking cadence-based standards [37, 38] have not been widely explored in large observational accelerometry studies. Devices such as the chest-mounted Actiheart (CamNTech Ltd, UK) featuring continuous combined heart rate monitoring and accelerometry can capture increased exertion associated with carrying loads and walking on different gradients. However, the uptake of such technology in large scale observational studies is unlikely due to the high price and participant burden (direct chest skin placement).

In summary, there is no established and validated method to capture frequency and duration of VILPA bouts in free-living conditions. Laboratory-derived VILPA measurement could be the first step, followed by their cross-validation within and between observational cohorts which are heterogenous with regards to cardiorespiratory fitness and other physiological markers known to respond to vigorous PA. Devices such as the Actiheart, which is validated against electrocardiogram (ECG) in both laboratory [39] and in free-living conditions [40], could be an appropriate validation standard for other accelerometry methods (e.g., wrist or thigh placements). Simple cadence-based standards to define vigorous intensity, such as the heuristic cut-off of 130 steps per minute [38], have a feasibility advantage as they can be used with all three most-common accelerometry monitoring locations (waist, wrist, thigh). The potential of device-based VILPA measurement standards will be fully realized once accelerometer manufacturers incorporate VILPA algorithms in their data processing and analysis software. For this reason, the empirical derivation and publication of such algorithms is a key research priority.

### Pillar 3: Health Effects

Recent proof-of concept trials in young adults suggested that as few as three 20-s VILPA-mimicking stair climbing bouts at > 85% of maximal heart rate (termed “exercise snacks”) on 3 days per week have measurable effects on cardiorespiratory fitness and aerobic power improvement in young adults within only 6 weeks [29, 41]. Such effects were evident both when the three daily bouts were performed with minimal recovery over a 10-min period or were interspersed 1–4 h apart [27]. Other trials have documented comparable effects using similar protocols [42, 43]. Such preliminary evidence is encouraging as it suggests that as little as 3 min of VILPA per week may stimulate the cardiorespiratory system and lead to measurable beneficial adaptations within 6 weeks. Another very small proof-of concept physiological trial in older adults with type 2 diabetes [44] demonstrated feasibility of the stair climbing intervention but found no effect on mean 24-h blood glucose over 6 weeks.

Future research would be significantly enhanced by studies that examine a broader range of VILPA activities. Depending on type of dwelling/workplace and geographical location, stair climbing and walking on a gradient could offer opportunities to attain a vigorous intensity stimulus for many. Modulation of intensity by walking pace [45], and/or weight carried (to mimic bodyweight interval training) is relatively easily achievable and can be standardized in free living. As alluded to above, there is a strong rationale to focus on those populations (i.e., physically inactive middle-aged and older adults with low CRF and/or other clinical risk factors) who are most likely to benefit from VILPA. With a few exceptions [46], most of the physiological trials summarized above were in younger healthy adults. Considering that adults in their early 20 s have not been exposed to physically inactive lifestyles for long enough, the relative CRF and aerobic power improvements achieved in many of the above studies [27, 29] may be greater in middle-aged or older populations due to the age accumulated physical inactivity related damage. Additionally, studies in middle-aged and older adults would be more readily translated into clinical and public health interventions and public health guidelines. Other important research gaps specific to older people exist. For example, no studies, to our knowledge, have examined how regular VILPA-like activity affects age-related functional decline.

Once acceptable device-based VILPA measurement standards are in place, large scale observational studies linked with surrogate (e.g., blood biomarkers) and long-term (e.g., cause-specific mortality and incident disease) outcomes will be an essential link to better understand the health effects of VILPA. Proof of concept trials with longer duration interventions would establish long-term efficacy for improving health outcomes, paving the way for better evidence-based behavioural interventions.

### Pillar 4: Behavioural Interventions

#### VILPA as an Intervention for Physically Inactive Middle-Aged and Older Adults

The majority of PA interventions are developed with a view to increasing continuous moderate to vigorous PA (MVPA). VILPA could be examined both as a stand-alone, and complementary to continuous MVPA, interventions. First, VILPA could be applied across a number of settings (work, home, leisure, etc.), and adapted for various physical, social and cultural contexts, and thus may have wider reach across different population groups. Assuming its theoretical feasibility advantage in some parts of the population (Fig. 2) will be confirmed, VILPA may be a promising intervention to facilitate sustained behaviour change [47]. Currently, limited evidence exists on how to most effectively promote VILPA. As a relatively unexplored PA pattern, an essential first step would be to assess the acceptability of VILPA in key population groups and develop appropriate messages and strategies to promote it. Multidisciplinary groups working with ends users could be employed to help refine and revise candidate strategies and messaging techniques to testing the clarity/readability, and relevance of messaging. Surveys and focus groups with end users would also provide opportunities to gauge feasibility, relevance and likelihood of engaging in VILPA. The outputs of such research could include mHealth tools (e.g., smartphone apps, text messaging), and other low-cost interventions to be delivered in community, primary care, and clinical settings. While sometimes the VILPA pattern could be facilitated by subtle changes in, e.g., the built environment, in some other occasions intervening to increase VILPA could involve conscious decisions for certain lifestyle changes, e.g., increase walking pace or use stairs instead of elevators. While VILPA is more closely integrated within daily living than, e.g., leisure-time physical activity, future research should be prepared to address some behaviour change challenges analogous to increasing continuous cumulative MVPA.

#### Consumer Wearable Activity Trackers and Smartphone Apps as VILPA-Promoting Tools

Technology-based PA approaches, such as wearable technologies (e.g., Fitbits) and smartphone apps, are prolific in both number and usage. Current offerings do not adequately address VILPA. For example, many apps offer guided planned exercise workouts (i.e., videos or instructions), or the ability to track workouts (e.g., GPS-based); workout-based PA is undertaken during leisure time, and typically in sustained bouts. Somewhat closer to VILPA are the short workout apps (e.g., 7-min workouts), however, these are still planned and structured. More in line with the incidental nature of VILPA are consumer wearable technologies and step-counting apps. Yet these products typically emphasise overall volume of activity (e.g., operationalised through a daily step goal) rather than accumulation of PA through intermittent bouts, and increase light-to-moderate rather than vigorous activity [48]. Many characteristics of wearable and smartphone technologies offer considerable promise for promoting VILPA. Apps may be used to deliver educational content (such as ideas for VILPA), track VILPA bouts (e.g., using the in-built accelerometer), and motivational messaging including prompts (e.g., using tailored push notifications). In addition, apps may be used to monitor real-time responses to bouts of VILPA (e.g., ecological momentary assessment of affective responses to VILPA). An example of an approach to be tested in the context of VILPA is the Just-In-Time Adaptive Interventions (JITAI), which aims to provide the stimulus to intervene when a person is likely to be most receptive [49]. Commonly, GPS technology is used to identify when an individual approach at-risk or opportune location [49]. A VILPA-specific JITAI intervention might, for example, suggest an individual take the stairs when they approach their office block on a workday, or suggest they carry a shopping basket rather than use a trolley when they are near a supermarket.

#### Specific Considerations for Promoting VILPA in Older Populations

Additional considerations apply to VILPA interventions aimed at older populations. The suitability and acceptability of incorporating VILPA in daily living would need to be evaluated with healthy older adults prior to focusing on clinical or at-risk populations of older adults. Assessment of risk also needs to be considered, including cardiovascular, respiratory and musculoskeletal. This would also allow the identification of acceptable strategies for facilitating VILPA without alarming older adults about potential health risks due to the physiological cues/responses linked with this exercise intensity (e.g., increased breathing, sudden increases in heart rate, and the need to move very fast and maintain balance) [50, 51]. Extensive consultation with older people about potential strategies and co-creation of a menu of appropriate approaches would be essential [52]. Further challenges may arise while implementing mHealth based interventions in older adults who are less familiar with technology.

Quick bursts of activity that do not require specific equipment/preparation/time investments may be particularly attractive to older adults, especially those who care for grandchildren where opportunities for VILPA may include carrying a child or playing with children [53]. Ensuring safety among people at higher risk of falls, or people with major musculoskeletal problems would be paramount.[54].

## Conclusions

We have provided an overview of the key issues and knowledge gaps to assist future research into defining, measuring and understanding the health effects of VILPA as an emerging strategy to achieve public health recommendations. Table 1 presents some *indicative* research priorities across the proposed four pillars of the VILPA framework. Recent progress with PA measurement capacities, mHealth tools, and changes in PA guidelines provide new opportunities to develop targeted interventions focusing on VILPA to improve population health. A key premise of the proposed research framework is that VILPA is a *complementary* and not a competing approach to traditional continuous MVPA, which is already well embedded in current PA guidelines and has been researched for several decades. Depending on the population and context, future interventions could test both stand-alone VILPA or VILPA combined with other more established PA approaches. An important outcome of such research efforts would be to provide members of the public, practitioners, and policy makers with new evidence to capitalize on this potentially feasible health-enhancing form of PA. We hope that the proposed framework will encourage research in this field, contribute to the alignment of research agendas of different research groups, and promote international multidisciplinary collaboration.

**Table 1:** Examples of future priorities in vigorous intermittent lifestyle physical activity (VILPA) research across the four-pillar framework

Pillar	Research priority
Definition	Standardise an empirically derived operational definition of VILPA for use in research and clinical practice Communicate a simple definition of VILPA with examples of key activities and duration of those activities, ensuring that this definition is clear to understand Adapt the definition for special groups, such as older people, where apparently simple low intensity activities may actually be VILPA in terms of relative intensity
Measurement	Develop and validate device-based measurements (accelerometers) that are sensitive enough to capture characteristics (e.g., duration and mode/type) of bouts of intermittent vigorous physical activity Develop and validate self-reported measurements that can capture daily frequency of vigorous intensity in different domains (e.g., work, transportation) Tailor measurement to research environment (e.g., large-scale observational studies, clinical trials) Explore the potential of consumer wearables for capturing VILPA bouts
Health effects	Explore the effects of VILPA on cardiorespiratory fitness, key cardiovascular and metabolic biomarkers in people at high risk of cardiovascular disease and the general middle aged and older adult population Explore population-wide health effects on key cardiometabolic, physical functioning, and quality of life outcomes using existing epidemiologic cohorts Explore if VILPA elicits similar health benefits to equivalent volumes of continuous moderate to vigorous intensity activity Explore if VILPA elicits complementary health benefits to other physical activity or sedentary behaviour reducing interventions
Behavioural interventions	Investigate the behavioural translation of VILPA into simple messages and behaviour change strategies targeting people at high risk of cardiovascular disease Explore the safety of regular and long-term VILPA in people at high risk of falls or major musculoskeletal conditions, especially older adults Explore capability, opportunities and motivation (e.g., attitudes, preferences) to engage in specific VILPA activities (i.e. COM-B system) Demonstrate proof of concept (e.g., can VILPA be maintained in the longer-term/over several months/years?) Co-create and evaluate the effectiveness of interventions specifically in middle aged and older adults Understand the real-time affective psychological responses to bouts of VILPA Develop time-tailored interventions using smartphone-based technologies

## Electronic supplementary material

Below is the link to the electronic supplementary material.

Supplementary file1 (DOCX 35 kb)

Supplementary file2 (DOCX 123 kb)

## Declarations

### Availability of data and material

Data transparency. Not applicable.

### Code availability

Software application or custom code. Not applicable.

## References

[1] Lee IM, Shiroma EJ, Lobelo F, Puska P, Blair SN, Katzmarzyk PT (2012). Effect of physical inactivity on major non-communicable diseases worldwide: an analysis of burden of disease and life expectancy. Lancet.

[2] World Health Organization. Global Recommendations on Physical Activity for Health. 2011. https://www.who.int/dietphysicalactivity/publications/recommendations18_64yearsold/en/. Accessed 28 Feb 2020.

[3] Guthold R, Stevens GA, Riley LM, Bull FC (2018). Worldwide trends in insufficient physical activity from 2001 to 2016: a pooled analysis of 358 population-based surveys with 1.9 million participants. Lancet Glob Health..

[4] Chau J, Chey T, Burks-Young S, Engelen L, Bauman A (2017). Trends in prevalence of leisure time physical activity and inactivity: results from Australian National Health Surveys 1989–2011. Aust N Z J Public Health.

[5] Hoare E, Stavreski B, Jennings GL, Kingwell BA (2017). Exploring motivation and barriers to physical activity among active and inactive Australian adults. Sports (Basel, Switzerland).

[6] Division of Nutrition, Physical Activity, and Obesity, National Center for Chronic Disease Prevention and Health Promotion. Perceived Exertion (Borg Rating of Perceived Exertion Scale). Centers for Disease Control and Prevention; 2020. https://bit.ly/3dGEbBa. Accessed 17 Oct 2020.

[7] Fletcher GF, Ades PA, Kligfield P, Arena R, Balady GJ, Bittner VA, Coke LA, Fleg JL, Forman DE, Gerber TC, Gulati M, Madan K, Rhodes J, Thompson PD, Williams MA (2013). Exercise standards for testing and training: a scientific statement from the American Heart Association. Circulation.

[8] Gebel K, Ding D, Chey T, Stamatakis E, Brown WJ, Bauman AE (2015). Effect of moderate to vigorous physical activity on all-cause mortality in middle-aged and older Australians. JAMA Intern Med.

[9] Rey Lopez JP, Gebel K, Chia D, Stamatakis E (2019). Associations of vigorous physical activity with all-cause, cardiovascular and cancer mortality among 64,913 adults. BMJ Open Sport Exerc Med.

[10] Lees FD, Clarkr PG, Nigg CR, Newman P (2005). Barriers to exercise behavior among older adults: a focus-group study. J Aging Phys Act.

[11] Loprinzi PD, Pariser G (2013). Cardiorespiratory fitness levels and its correlates among adults with diabetes. Cardiopulm Phys Ther J.

[12] Australian Institute of Health and Welfare. Australia's health 2018. 2018. https://www.aihw.gov.au/reports/australias-health/australias-health-2018-in-brief/contents/about. Accessed 28 Feb 2020.

[13] National Heart Foundation of Australia. Australian heart disease statistics. Overweight, obesity and cardiovascular disease—past, present and future. 2015. https://www.heartfoundation.org.au/about-us/what-we-do/heart-disease-in-australia/australian-heart-disease-statistics. Accessed 28 Feb 2020.

[14] Bollyky TJ, Templin T, Cohen M, Dieleman JL (2017). Lower-income countries that face the most rapid shift in noncommunicable disease burden are also the least prepared. Health Aff (Millwood).

[15] O'Donovan G, Lee IM, Hamer M, Stamatakis E (2017). Association of "Weekend Warrior" and other leisure time physical activity patterns with risks for all-cause, cardiovascular disease, and cancer mortality. JAMA Intern Med.

[16] Evenson KR, Wen F, Herring AH (2016). Associations of accelerometry-assessed and self-reported physical activity and sedentary behavior with all-cause and cardiovascular mortality among US adults. Am J Epidemiol.

[17] Stamatakis E, Johnson N, Powell L, Hamer M, Rangul V, Holtermann A (2018). Short and sporadic bouts in the 2018 US Physical Activity Guidelines: could high intensity incidental physical activity be the new HIIT?. Br J Sports Med.

[18] Piercy KL, Troiano RP, Ballard RM (2018). The physical activity guidelines for Americans. JAMA.

[19] UK Chief Medical Officers. UK Chief Medical Officers' Physical Activity Guidelines. 2019. https://www.whitwellprimary.co.uk/uk-chief-medical-officers-physical-activity-guidel/. Accessed 28 Feb 2020.

[20] Thompson D, Batterham AM (2013). Towards integrated physical activity profiling. PLoS ONE.

[21] Thompson D, Peacock O, Western M, Batterham AM (2015). Multidimensional physical activity: an opportunity, not a problem. Exerc Sport Sci Rev.

[22] Department of Health. Australia’s Physical Activity and Sedentary Behaviour Guidelines for Adults (18–64 years). 2014. https://www1.health.gov.au/internet/main/publishing.nsf/Content/health-pubhlth-strateg-phys-act-guidelines. Accessed 28 Feb 2020.

[23] Australian Institute of Health and Welfare. Impact of physical inactivity as a risk factor for chronic conditions. Canberra: AIHW: Australian Burden of Disease Study. 2017. https://www.aihw.gov.au/reports/burden-of-disease/impact-of-physical-inactivity-chronic-conditions/contents/table-of-contents. Accessed 28 Feb 2020.

[24] Batacan RB, Duncan MJ, Dalbo VJ, Tucker PS, Fenning AS (2017). Effects of high-intensity interval training on cardiometabolic health: a systematic review and meta-analysis of intervention studies. Br J Sports Med.

[25] Biddle SJ, Batterham AM (2015). High-intensity interval exercise training for public health: a big HIT or shall we HIT it on the head?. Int J Behav Nutr Phys.

[26] Zenko Z, Ekkekakis P, Ariely D (2016). Can you have your vigorous exercise and enjoy it too? Ramping intensity down increases postexercise, remembered, and forecasted pleasure. J Sport Exerc Psychol.

[27] Jenkins EM, Nairn LN, Skelly LE, Little JP, Gibala MJ (2019). Do stair climbing exercise “Snacks” improve cardiorespiratory fitness?. Appl Physiol Nutr Med.

[28] Francois ME, Baldi JC, Manning PJ, Lucas SJ, Hawley JA, Williams MJ (2014). 'Exercise snacks' before meals: a novel strategy to improve glycaemic control in individuals with insulin resistance. Diabetologia.

[29] Allison MK, Baglole JH, Martin BJ, Macinnis MJ, Gurd BJ, Gibala MJ (2017). Brief intense stair climbing improves cardiorespiratory fitness. Med Sci Sports Exerc.

[30] Stamatakis E, Straker L, Hamer M, Gebel K (2019). The 2018 Physical Activity Guidelines for Americans: what's new? Implications for clinicians and the public. J Orthop Sports Phys Ther.

[31] Murphy MH, Lahart I, Carlin A, Murtagh E. The effects of continuous compared to accumulated exercise on health: a meta-analytic review. Sports Med (Auckland, NZ). 2019;1–23. 10.1007/s40279-019-01145-2 PMC6745307 31267483

[32] Physical Activity Guidelines Advisory Committee. 2018 Physical Activity Guidelines Advisory Committee Scientific Report. 2018. https://health.gov/our-work/physical-activity/current-guidelines/scientific-report. Accessed 28 Feb 2020.

[33] Skotte J, Korshoj M, Kristiansen J, Hanisch C, Holtermann A (2014). Detection of physical activity types using triaxial accelerometers. J Phys Act Health.

[34] Loprinzi PD, Lee H, Cardinal BJ, Crespo CJ, Andersen RE, Smit E (2012). The relationship of actigraph accelerometer cut-points for estimating physical activity with selected health outcomes: results from NHANES 2003–2006. Res Q Exerc Sport.

[35] Hamer M, Stamatakis E, Chastin S, Pearson N, Brown M, Gilbert E (1970). Feasibility of measuring sedentary time with thigh worn accelerometry, and sociodemographic correlates: the 1970 British Cohort Study. Am J Epidemiol.

[36] Saint-Maurice PF, Troiano RP, Bassett DR, Graubard BI, Carlson SA, Shiroma EJ (2020). Association of daily step count and step intensity with mortality among US adults. JAMA.

[37] Tudor-Locke C, Han H, Aguiar EJ, Barreira TV, Schuna JM, Kang M (2018). How fast is fast enough? Walking cadence (steps/min) as a practical estimate of intensity in adults: a narrative review. Br J Sports Med.

[38] Tudor-Locke C, Aguiar EJ, Han H, Ducharme SW, Schuna JM, Barreira TV (2019). Walking cadence (steps/min) and intensity in 21–40 year olds: CADENCE-adults. Int J Behav Nutr Phys..

[39] Brage S, Brage N, Ekelund U, Luan J, Franks PW, Froberg K (2006). Effect of combined movement and heart rate monitor placement on physical activity estimates during treadmill locomotion and free-living. Eur J Appl Physiol.

[40] Watson KB, Carlson SA, Carroll DD, Fulton JE (2014). Comparison of accelerometer cut points to estimate physical activity in US adults. J Sports Sci.

[41] Little JP, Langley J, Lee M, Myette-Côté E, Jackson G, Durrer C (2019). Sprint exercise snacks: a novel approach to increase aerobic fitness. Eur J Appl Physiol.

[42] Gillen JB, Martin BJ, MacInnis MJ, Skelly LE, Tarnopolsky MA, Gibala MJ (2016). Twelve weeks of sprint interval training improves indices of cardiometabolic health similar to traditional endurance training despite a fivefold lower exercise volume and time commitment. PLoS ONE.

[43] Vollaard NBJ, Metcalfe RS (2017). Research into the health benefits of sprint interval training should focus on protocols with fewer and shorter sprints. Sports Med (Auckland, NZ).

[44] Godkin FE, Jenkins EM, Little JP, Nazarali Z, Percival ME, Gibala MJ (2018). The effect of brief intermittent stair climbing on glycemic control in people with type 2 diabetes: a pilot study. Appl Physiol Nutr Med.

[45] Stamatakis E, Kelly P, Strain T, Murtagh EM, Ding D, Murphy MH (2018). Self-rated walking pace and all-cause, cardiovascular disease and cancer mortality: individual participant pooled analysis of 50,225 walkers from 11 population British cohorts. Br J Sports Med.

[46] Metcalfe RS, Fitzpatrick B, Fitzpatrick S, McDermott G, Brick N, McClean C (2018). Extremely short duration interval exercise improves 24-h glycaemia in men with type 2 diabetes. Eur J Appl Physiol.

[47] Kwasnicka D, Dombrowski SU, White M, Sniehotta F (2016). Theoretical explanations for maintenance of behaviour change: a systematic review of behaviour theories. Health Psychol Rev.

[48] Maher C, Ferguson M, Vandelanotte C, Plotnikoff R, De Bourdeaudhuij I, Thomas S (2015). A web-based, social networking physical activity intervention for insufficiently active adults delivered via Facebook app: randomized controlled trial. J Med Internet Res.

[49] Nahum-Shani I, Smith SN, Spring BJ, Collins LM, Witkiewitz K, Tewari A (2017). Just-in-time adaptive interventions (JITAIs) in mobile health: key components and design principles for ongoing health behavior support. Ann Behav Med.

[50] Burton E, Farrier K, Lewin G, Pettigrew S, Hill A-M, Airey P (2017). Motivators and barriers for older people participating in resistance training: a systematic review. J Aging Phys Act.

[51] Franco MR, Tong A, Howard K, Sherrington C, Ferreira PH, Pinto RZ (2015). Older people’s perspectives on participation in physical activity: a systematic review and thematic synthesis of qualitative literature. Br J Sports Med.

[52] Stathi A, Western M, De Koning J, Perkin O, Withall J, Nyman S (2018). Implementing physical activity programmes for community-dwelling older people with early signs of physical frailty. The Palgrave handbook of ageing and physical activity promotion.

[53] Ainsworth BE, Haskell WL, Herrmann SD, Meckes N, Bassett DR, Tudor-Locke C (2011). Compendium of physical activities: a second update of codes and MET values. Med Sci Sports Exerc.

[54] García-Hermoso A, Ramirez-Vélez R, Sáez de Asteasu ML, Martínez-Velilla N, Zambom-Ferraresi F, Valenzuela PL (2020). Safety and effectiveness of long-term exercise interventions in older adults: a systematic review and meta-analysis of randomized controlled trials. Sports Med..

[55] Martenstyn JA, Powell L, Nassar N, Hamer M, Stamatakis E (2019). Intensity-weighted physical activity volume and risk of all-cause and cardiovascular mortality: does the use of absolute or corrected intensity matter?. J Phys Act Health.

[56] Stamatakis E, Johnson N, Powell L, Hamer M, Rangul V, Holtermann A (2019). Short and sporadic bouts in the 2018 US Physical Activity Guidelines: could high intensity incidental physical activity be the new HIIT?. Br J Sports Med.

