# Supplementary material for: Untapping the Health Enhancing Potential of Vigorous Intermittent Lifestyle Physical Activity (VILPA): Rationale, Scoping Review, and a 4-Pillar Research Framework
Source: Sports Med. 2020 Oct 26;51(1):1–10. doi: 10.1007/s40279-020-01368-8 (PMC7806564; doi:10.1007/s40279-020-01368-8)
Supplement: Supplementary file 1 — Supplementary file1 (DOCX 35 kb) [file 40279_2020_1368_MOESM1_ESM.docx]

**Article title**

Untapping the health enhancing potential of vigorous intermittent lifestyle physical activity (VILPA): rationale, scoping review, and a 4-pillar research framework

**Journal name**

Sports Medicine

**Author names**

Emmanuel Stamatakis, Bo-Huei Huang, Carol Maher, Cecilie Thøgersen-Ntoumani, Afroditi Stathi, Paddy C. Dempsey, Nathan Johnson, Andreas Holtermann, Josephine Y. Chau, Catherine Sherrington, Amanda J Daley, Mark Hamer, Marie H Murphy, Tudor-Locke Catrine, Martin J Gibala

**Affiliation and e-mail address of the corresponding author**

Emmanuel Stamatakis

University of Sydney, Charles Perkins Centre, Faculty of Medicine and Health, School of Health SciencesSydney, Australia

E-mail address: emmanuel.stamatakis@sydney.edu.au

**Captions**

Results of the scoping review.

**Declarations**

**Funding**

This work was partly funded through a National Health and Medical Research Council (NHMRC) Ideas Grant (#APP1180812). ES is funded by an NHMRC Senior Research Fellowship (#APP1110526). CS is funded by an NHMRC Senior Research Fellowship (#APP1079267). CM is funded by an NHMRC Career Development Award Fellowship (#APP1125913). PCD is supported by a NHMRC Fellowship (#APP1142685) and the UK Medical Research Council (#MC_UU_12015/3). MJG is supported by the Natural Sciences and Engineering Research Council (# RGPIN-2015-04632). AJD is funded by a NIHR Research Professorship award.

**Conflicts of interest/Competing interests** (include appropriate disclosures)

None

**Availability of data and material** (data transparency)

Not applicable

**Code availability** (software application or custom code)

Not applicable

**SUPPLEMENT 1 – SCOPING REVIEW METHODS**

We conducted the searches in December 2019, using PubMed, CINAHL, and Embase with timeframe starting from inception to December 19th. The search strategy (shown below) and methods adhered to the PRISMA reporting standard and extension for scoping reviews [1, 2]. After removing duplicates, articles were screened for eligibility in two sequential stages by one reviewer. The first stage involved a title and abstract screening, and articles not aligned with our PICOS (shown below) were excluded. The second stage involved a full-text review and the reason for each excluded article was noted (see study exclusion flowchart in Online Resource 2). All stages of study selection were carried out by one reviewer (co-author B-H H).

We searched the literature for peer-reviewed studies (including reviews) in human participants. To be considered as eligible for inclusion, studies had to address at least one of the relevant areas of any concept relating to vigorous intermittent lifestyle physical activity (VILPA): a) definition, b) measurement, c) health effects, and d) foundations for behavioural interventions. Observational studies were excluded if the intensity and time of incidental physical activity was not specified; interventional studies were not included if the intervention required ‘all-out’ efforts. Besides, studies were excluded if they were: a) not written in English, b) full text unavailable, or c) commentary or editorial.

**Search Strategy: PubMed**

Database: PubMed; Date of Search: 19 December 2019; Results: 102

| **Set** | **Search Strategy** |
| --- | --- |
| VILPA -related concepts | (  ("High intensity"[tiab] OR "High-intensity"[tiab] OR “Vigorous"[tiab] OR “Strenuous"[tiab] OR “Intense"[tiab] OR “All-out"[tiab])  AND  (“Physical activity"[tiab] OR “Physical activities”[tiab] OR "Activity bout"[tiab] OR “Activity session"[tiab] OR “Exercise"[tiab] OR “Burst"[tiab])  AND  (“Sporadic”[tiab] OR “Snack”[tiab] OR “Incidental”[tiab] OR “Daily Living”[tiab])  ) |
| Limit: Publication Type Exclude Commentaries/Editorials | NOT (“comment”[Publication Type] OR “editorial”[Publication Type]) |
| Limit: Language | AND (English[lang]) |
| Limit: Exclude animal only | NOT ("Animals"[Mesh]) NOT ("Animals"[Mesh] AND "Humans"[Mesh]) |

**Search Strategy: CINAHL**

Database: CINAHL; Date of Search: 19 December 2019; Results: 139

Terms searched in title or abstract

| **Set** | **Search Strategy** |
| --- | --- |
| VILPA | (  (AB ("High intensity" OR "High-intensity" OR “Vigorous" OR “Strenuous" OR “Intense" OR “All-out") OR TI ("High intensity" OR "High-intensity" OR “Vigorous" OR “Strenuous" OR “Intense" OR “All-out"))  AND  (AB (“Physical activity" OR “Physical activities” OR "Activity bout"  OR “Activity session OR “Exercise” OR “Bust”) OR TI (“Physical activity" OR “Physical activities” OR "Activity bout" OR “Activity session OR “Exercise” OR “Bust”))  AND  (AB (“Sporadic” OR “Snack” OR “Incidental” OR “Daily living” OR “Time efficient”) OR TI (“Sporadic” OR “Snack” OR “Incidental” OR “Daily living” OR “Time efficient”))  ) |
| Limits | English language  Peer reviewed  Human  All years searched |

**Search Strategy: Embase**

Database: Embase; : 19 December 2019; Results: 92

| **Set** | **Search Strategy** |
| --- | --- |
| VILPA | (  (High*intensity OR High-intensity OR Vigorous OR Strenuous OR Intense OR All-out)  AND  (Physical*activity OR Physical*activities OR Activity*bout OR Activity*session OR Exercise OR Burst)  AND  (Sporadic OR Snack OR Incidental OR Daily*living OR Time*efficient)  ).ab,ti. |
| Limits | English language  Human  All years searched |

**PICOS (participants, interventions, comparators, outcomes, and study design)**

**Participants:**

There were no age or sex restrictions.

**Interventions:**

For the interventional study, the exercise program must 1) not require ‘all-out’ efforts; 2) indicate its frequency, intensity, time, and type (FITT). For long-term intervention, the progression (if applicable) should be indicated.

**Comparator:**

For the interventional study, a control group with either no intervention or different exercise intervention is eligible. A pre-post study without control group is also eligible.

**Outcomes:**

For the observational study, a physical activity measurement monitoring both intensity and time is required, while no other outcomes are necessary.

For the interventional study, an outcome representing any aspects (*e.g*., cardiovascular fitness, cardiometabolic risk factors) of health effects is required.

**Study design:**

For both the observational and interventional studies, all study designs are eligible (*i.e*., observational study: ecological, proportional mortality, case-crossover, cross-sectional, case-control, and retrospective and prospective cohort; interventional study: pre-post, quasi-experiment, and randomized controlled trial).

**REFERENCES**

1. Moher D, Liberati A, Tetzlaff J, Altman DG, Group P. Preferred reporting items for systematic reviews and meta-analyses: the PRISMA statement. J Clin Epidemiol. 2009;62(10):1006-12. doi:10.1016/j.jclinepi.2009.06.005.

2. Tricco AC, Lillie E, Zarin W, O'Brien KK, Colquhoun H, Levac D et al. PRISMA Extension for Scoping Reviews (PRISMA-ScR): Checklist and Explanation. Ann Intern Med. 2018;169(7):467-73. doi:10.7326/M18-0850.
