# Supplementary material for: Untapping the Health Enhancing Potential of Vigorous Intermittent Lifestyle Physical Activity (VILPA): Rationale, Scoping Review, and a 4-Pillar Research Framework
Source: Sports Med. 2020 Oct 26;51(1):1–10. doi: 10.1007/s40279-020-01368-8 (PMC7806564; doi:10.1007/s40279-020-01368-8)
Supplement: Supplementary file 2 — Supplementary file2 (DOCX 123 kb) [file 40279_2020_1368_MOESM2_ESM.docx]

**Article title**

Untapping the health enhancing potential of vigorous intermittent lifestyle physical activity (VILPA): rationale, scoping review, and a 4-pillar research framework

**Journal name**

Sports Medicine

**Author names**

Emmanuel Stamatakis, Bo-Huei Huang, Carol Maher, Cecilie Thøgersen-Ntoumani, Afroditi Stathi, Paddy C. Dempsey, Nathan Johnson, Andreas Holtermann, Josephine Y. Chau, Catherine Sherrington, Amanda J Daley, Mark Hamer, Marie H Murphy, Tudor-Locke Catrine, Martin J Gibala

**Affiliation and e-mail address of the corresponding author**

Emmanuel Stamatakis

University of Sydney, Charles Perkins Centre, Faculty of Medicine and Health, School of Health SciencesSydney, Australia

E-mail address: emmanuel.stamatakis@sydney.edu.au

**Captions**

Results of the scoping review.

**Declarations**

**Funding**

This work was partly funded through a National Health and Medical Research Council (NHMRC) Ideas Grant (#APP1180812). ES is funded by an NHMRC Senior Research Fellowship (#APP1110526). CS is funded by an NHMRC Senior Research Fellowship (#APP1079267). CM is funded by an NHMRC Career Development Award Fellowship (#APP1125913). PCD is supported by a NHMRC Fellowship (#APP1142685) and the UK Medical Research Council (#MC_UU_12015/3). MJG is supported by the Natural Sciences and Engineering Research Council (# RGPIN-2015-04632). AJD is funded by a NIHR Research Professorship award.

**Conflicts of interest/Competing interests** (include appropriate disclosures)

None

**Availability of data and material** (data transparency)

Not applicable

**Code availability** (software application or custom code)

Not applicable

**SUPPLEMENT 2 - RESULTS OF THE SCOPING REVIEW & BRIEF COMMENT**

As shown in **Figure S1** below, of the 333 identified records, 23 were removed because of duplication; 254 were further excluded after the title and abstract screening; another 49 were excluded after the full-text screening. Two studies were identified through manual citation searching. In total, four cross-sectional studies [1-4], one narrative review [5], and four interventions [6-9] were included in the present scoping review. Study details, definition/measurement of the VILPA-relevant concept, and results of the observational and intervention studies were extracted (see **Tables S1-2** for details of all identified studies).

**Characteristics and results of observational studies**

All the four observational studies used the ActiGraph accelerometer to measure and classify vigorous PA while only one study [1] involved adult participants and the rest [2-4] were in children. In an analysis of the 2003–2006 U.S. National Health and Examination Nutrition Survey, Robson and Janssen (2015) suggested that the median daily volume of incidental vigorous PA (≥ 5,999 counts/min) was 0 (interquartile range: 0-0) (min/d) among adult participants with different age, race/ethnicity, and body mass index [1]. The median daily volume remained zero (Interquartile range: 0.0-0.2) when they further incorporated sporadic vigorous PA bout. Although children spent more time (> 10 min/d) in incidental vigorous PA with most bout duration less than 15 seconds [2-4], associations of incidental vigorous PA with health outcomes were unclear. In a small cross-sectional analysis (n = 47), Stone et al. (2009) indicated that both daily incidental vigorous PA (> 6,130 counts/min) and “hard” PA (> 9,630 counts/min) volumes were associated with lower waist circumference and higher cardiovascular fitness [2]. Chinapaw et al. (2019) suggested that neither frequency nor volume of incidental vigorous PA (≥ 4,012 counts/min) differed by obesity status in a cross-sectional study (n = 1,218) [4].

The definition of vigorous PA in observational studies was largely restricted to the capacity of measurements, *i.e*., recording epoch and accelerometry types. Robson and Janssen (2015) used an one-minute recording epoch and showed almost no daily incidental vigorous PA in U.S. adults[1]. Devices with shorter epochs (*e.g.,* 10 seconds or less) may be better able to pick up very short sporadic PA and therefore may provide a more detailed vigorous PA profile [2-5]. However, this does not explain the extremely low median VILPA values noted above [1]. Besides accelerometer epoch, accelerometry type (such as uniaxial pedometer) could also underestimate relative PA intensity, especially within short bursts [5].

In conclusion, very little data on VILPA and health outcomes in children exists, while adults’ VILPA in daily life cannot be captured with previous generation accelerometry (waist worn, one-minute epochs).

**Characteristics and results of intervention studies**

All the four included interventional studies focused on the chronic effects of a regular VILPA relevant intervention for more than six weeks [6-9]. All studies applied a ~90% HR_max_ intensity exercise protocol, with stair climbing as exercise mode in three and fasting walking in one.

Regular VILPA-like stair-climbing intervention, three times per week for six to eight weeks, consistently improved cardiovascular fitness (VO_2max_) [6, 8]. Boreham et al. (2005) suggested that a single bout of 2-min stair climbing at 199 step/min (~90% HR_max_) could improve VO_2ma_ and reduce low-density lipoprotein cholesterol among young adult (19 yr) [6]. The potential benefit of VILPA-like intervention on glucose metabolism was observed in one study of mid-aged healthy adults (48 yr) [7], but not in young healthy adults or mixed-aged diabetes patients [8, 9].

In conclusion, existing VILPA-like interventions are proof-of-concept in nature. This preliminary evidence suggests that brief bouts of vigorous-intensity physical activity (stair climbing or fast walking) can improve cardiovascular fitness with around 10-minute non-consecutive vigorous exercise per day. The limited available evidence also suggested that intermittent vigorous physical activity may have favourable affective responses, compared to continuous exercise.

**Narrative review**

Rowlands and Eston (2007) narratively reviewed studies using device-based measurements to assess and interpret children’s physical activity [5]. Similar to the characteristics of VILPA in the present scoping review; the authors suggested that the activity pattern in children is intermittent, comprising short and frequent bouts. The authors concluded that when both intensity/pattern and total amount are of interest, accelerometry is a better measurement tool, compared to pedometer. A hybrid of accelerometry and heart rate monitor (such as Actiheart) was also suggested to better estimate energy expenditure when applicable.


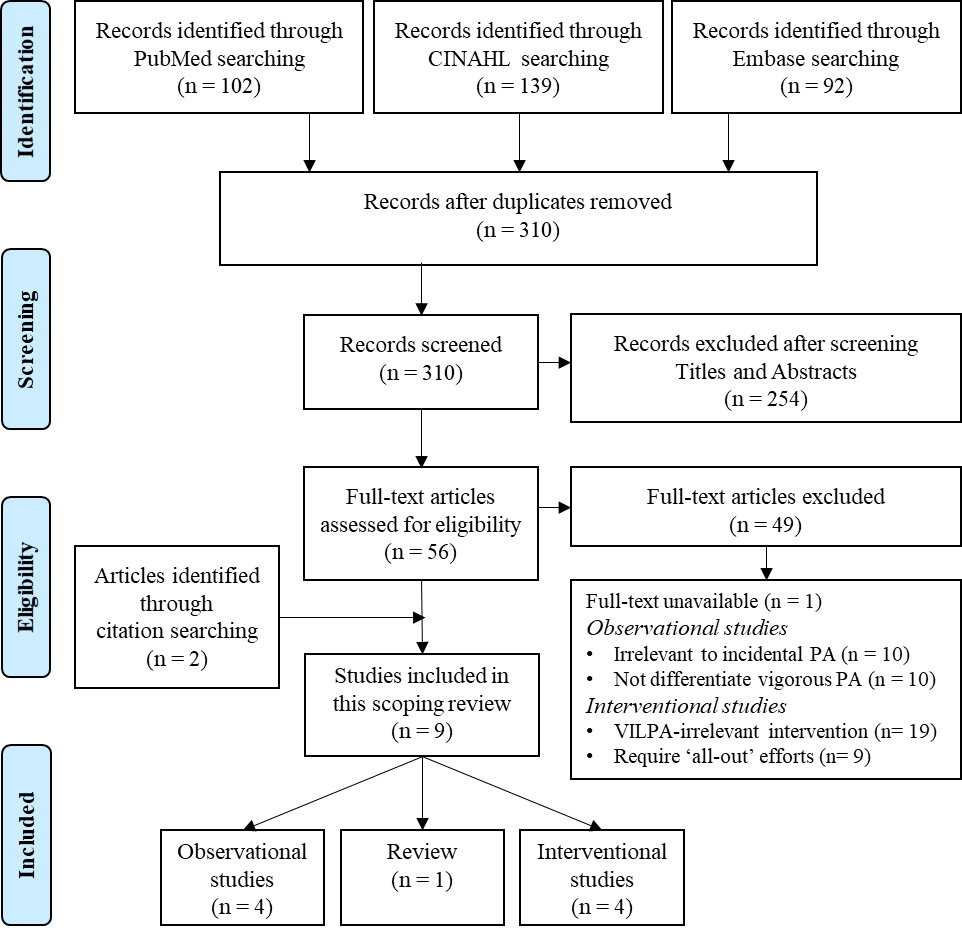


**Fig. S1 PRISMA flow of the present scoping review.**

**Table S1. Details of the included observational studies.**

| **Author(s)** | **Study Details**   1. Design 2. Years 3. Sampling method 4. Multi-centre? 5. N and characteristic 6. Age 7. Gender 8. Setting (community, occupational, clinical, other) 9. Study Type (descriptive; health outcomes; correlates) 10. Mother study name | **Definition** | **Measurement** | **Results** |
| --- | --- | --- | --- | --- |
| Stone et al. (2009) | 1. Cross sectional 2. 2006 – 2007 3. Convenience sampling 4. Multi centre 5. N = 47 healthy children 6. Mean = 9.2 yr 7. Male 8. Community 9. Correlates 10. -na | Vigorous PA (> 6 METs, > 6,130 counts/min) in bouts (≥ 4 s and < 5 min).  Hard PA (> 9,630 counts/min) in bouts (≥ 4 s and < 5 min) | ActiGraph GT1M accelerometer  (Pensacola, FL) with 2-s recording epoch for seven consecutive days. | Average vigorous PA duration: 5.5 (s), with frequency: 58.4 (times/d) and volume: 8.6 (min/d).  Average hard PA duration: 6 (s), with frequency: 9.8 (times/d) and volume: 2.6 (min/d).  Both vigorous and hard PA were correlated to waist circumference and cardiovascular fitness. |
| Robson and Janssen (2015) | 1. Cross sectional 2. 2003 – 2006 3. Nationally representative purposive sampling 4. Multi centre 5. N = 6,040 adults 6. Over 20 yr 7. Both Female and Male 8. Other 9. Descriptive 10. the U.S. National Health and Nutrition Examination Survey (NHANES) | Vigorous PA (≥ 5,999 counts/min) in bouts < 10 min or in bouts embedded in ≥ 10 min light PA bouts. | ActiGraph AM-7164  uniaxial accelerometers (Pensacola, FL) with 1-min recording epoch for seven consecutive days. | Medium volume of both independent and embedded vigorous PA: 0 (min/d) among participants with different age, race/ethnicity, and body mass index. |
| Downs et al.  (2017) | 1. Cross sectional 2. 2013 3. Convenience sampling 4. Multi centre 5. N = 38 children and adolescent with intellectual disabilities 6. Mean = 9.97 yr 7. Both Female and Male 8. Community 9. Correlates 10. -na | Vigorous PA (≥ 4,012 counts/min) in bouts ≥ 5, 10, 15, 30, 60, and 180 s. | ActiGraph GT1M accelerometer  (Pensacola, FL) with 5-s recording epoch for seven consecutive days. | No difference in vigorous PA time between sexes.  Average vigorous PA volume: 19.5 (min/d) with 91% vigorous PA bouts accumulated in bouts <15s while none was accumulated in bouts over 180 s.  Participants with intellectual disabilities showed lower PA levels. |
| Chinapaw et al. (2019) | 1. Cross sectional 2. 2007 3. Convenience sampling 4. Multi centre 5. N = 1,218 healthy children 6. 5.5 - 12 yr 7. Both Female and Male 8. Community 9. Descriptive 10. CHAMPS study DK | Vigorous PA (≥ 4,012 counts/min) in bouts < 5 min, 5-9.9 min, and ≥ 10 min. | ActiGraph GT3X accelerometer (Pensacola, FL) with 2-s recording epoch for seven consecutive days. | Average vigorous PA in bouts (< 5 min) frequency: 103 (times/d) and volume: 43.1 (min/d).  Average vigorous PA in bouts (5-9.9 min) frequency: 0.02 (times/d) and volume: 0.06 (min/d).  Average vigorous PA in bouts (≥ 10 min) frequency: 0.005 (times/d) and volume: 0.01 (min/d).  Frequency and volume with all definitions showed no differences between overweight status. |

**Table S2. Details of the included interventional studies.**

| **Author(s)** | **Study Details**   1. Design 2. N and characteristic 3. Age 4. Gender | **Intervention** | | | | **Results** |
| --- | --- | --- | --- | --- | --- | --- |
|  |  | Mode | Intensity & Design | Session  time  (min)* | Frequency  & Total duration |  |
| Boreham  et al.  (2005) | 1. Randomized controlled trial 2. 15 healthy sedentary adults 3. 18.8 ± 0.7 4. 15 women | Stair climbing | Intervention: 1 X 199-step (32.8-m) vigorous (90 step/min) climbing  Control: No exercise | ~2 | 2-5/d  5/wk  8 wk | VO_2max_ ↑  low-density lipoprotein cholesterol ↓  No effect on BMI, homocysteine and other lipid profile. |
| Francois et al. (2014) | 1. Randomized controlled trial 2. 9 healthy adults 3. 48 ± 6 4. 7 men; 2 women | Treadmill | Intervention: 6 X 1-min walk (90% HR_max_)/ 1-min recovery (slow walk)  Control: 1 X 30-min walk (60% HR_max_) | 11 | 3/d  5/wk  1 wk | 3-h postprandial glucose after breakfast and dinner, 24-h mean glucose concentration ↓  No effect on 3-h postprandial glucose after lunch. |
| Allison et al. (2017) | 1. Pre-post 2. 11 healthy sedentary adults 3. 26 ± 11 4. 11 women | Stair climbing | Intervention:  3 X 60-s vigorous (up to ~90% HR_max_) stair climbing up and casually down/ 1-mim recovery  OR  3 X 60-s vigorous (up to ~90% HR_max_) stair climbing up and casually down by 2 flights/ 1-mim recovery | 5 | 3/wk  6 wk | VO_2max_, peak power output, BMI, body mass, fat-free mass↑  No effect on fat mass, % body fat, blood pressure, mean glucose, insulin concentrations, AUC for glucose and insulin, fasting glucose and insulin concentrations, HOMA-IS, ISI-Cederholm. |
| Godkin et al. (2018) | 1. Pre-post 2. 7 type 2 diabetes patients 3. 21-70 4. 5 men; 2 women | Stair climbing | Session: 3 X 1-min vigorous (up to ~90% HR_max_) stair climbing up and casually down/ 1-min walk recovery | 5 | 3/wk  6 wk | Immediately blood glucose ↓.  No effect on mean 24-h glucose, time spent in hyperglycemia, glycemic variability, mean amplitude of glycemic excursion, postprandial glucose, fasting insulin, insulin sensitivity. |

*Time spent in warm-up and cool-down was not included in the session time.

**REFERENCES**

1. Robson J, Janssen I. A description of the volume and intensity of sporadic physical activity among adults. BMC Sports Sci Med Rehabil. 2015;7:2. doi:10.1186/2052-1847-7-2.

2. Stone MR, Rowlands AV, Middlebrooke AR, Jawis MN, Eston RG. The pattern of physical activity in relation to health outcomes in boys. International Journal of Pediatric Obesity. 2009;4(4):306-15. doi:10.3109/17477160902846179.

3. Downs SJ, Fairclough SJ, Knowles ZR, Boddy LM. Physical Activity Patterns in Youth With Intellectual Disabilities. Adapted Physical Activity Quarterly. 2016;33(4):374-90. doi:10.1123/APAQ.2015-0053.

4. Chinapaw MJ, Altenburg TM, Wang X, Andersen LB. From Total Volume to Sequence Maps: Sophisticated Accelerometer Data Analysis. Medicine & Science in Sports & Exercise. 2019;51(4):814-20. doi:10.1249/MSS.0000000000001849.

5. Rowlands AV, Eston RG. The Measurement and Interpretation of Children's Physical Activity. J Sports Sci Med. 2007;6(3):270-6.

6. Boreham C, Kennedy R, Murphy M, Tully M, Wallace W, Young I. Training effects of short bouts of stair climbing on cardiorespiratory fitness, blood lipids, and homocysteine in sedentary young women. British journal of sports medicine. 2005;39(9):590-3.

7. Francois ME, Baldi JC, Manning PJ, Lucas SJ, Hawley JA, Williams MJ et al. ‘Exercise snacks’ before meals: a novel strategy to improve glycaemic control in individuals with insulin resistance. Diabetologia. 2014;57(7):1437-45.

8. Allison MK, Baglole JH, Martin BJ, Macinnis MJ, Gurd BJ, Gibala MJ. Brief Intense Stair Climbing Improves Cardiorespiratory Fitness. Med Sci Sports Exerc. 2017;49(2):298-307. doi:10.1249/MSS.0000000000001188.

9. Godkin FE, Jenkins EM, Little JP, Nazarali Z, Percival ME, Gibala MJ. The effect of brief intermittent stair climbing on glycemic control in people with type 2 diabetes: a pilot study. Appl Physiol Nutr Metab. 2018;43(9):969-72. doi:10.1139/apnm-2018-0135.
